# A cross-sectional analysis of intimate partner violence and family planning use in rural India

**DOI:** 10.1016/j.eclinm.2020.100318

**Published:** 2020-04-18

**Authors:** Grace L. Chen, Jay G. Silverman, Anvita Dixit, Shahina Begum, Mohan Ghule, Madhusudana Battala, Nicole E. Johns, Anita Raj, Sarah Averbach

**Affiliations:** a University of California San Diego School of Medicine, 9500 Gilman Drive #0606, La Jolla, CA 92093, United States; b Center on Gender Equity and Health, University of California San Diego School of Medicine, 9500 Gilman Drive #0507, La Jolla, CA, 92093, United States; c Joint Doctoral Program in Public Health, San Diego State University, University of California San Diego, San Diego, CA, United States; d Department of Biostatistics, ICMR-National Institute for Research in Reproductive Health, J.M Street, Parel, Mumbai 400012, India; e Population Council, Zone 5A, Ground Floor, India Habitat Center, Lodi Road, New Delhi 110003, India; f Department of Obstetrics, Gynecology, and Reproductive Sciences, University of California San Diego School of Medicine, 9300 Campus Point Drive #7433, La Jolla, CA 92037, USA

**Keywords:** Intimate partner violence (IPV), Contraception, Family planning, Reproductive health, India

## Abstract

**Background:**

Intimate partner violence (IPV) has been shown to be associated differentially with contraceptive use based on type, with IPV more likely among pill users and less likely among condom users. Recent increases in IUD uptake allow consideration of this type of contraceptive. We assessed the association between self-reported IPV and self-reported contraceptive use, by type, among non-pregnant married women in rural India in a region with higher than average IUD use.

**Methods:**

We assessed the association between past 12-month IPV (physical, sexual, or any) and past 3-month contraceptive use (condom, pill, IUD, or any modern method) using crude and adjusted multinomial logistic regression models.

**Findings:**

Among the 1001 women included, 109 (10·9%) reported experiencing physical IPV and 27 (2·7%) reported experiencing sexual IPV in the past 12 months. Women experiencing physical IPV were significantly less likely to use condoms (adjusted relative risk ratio [RRR]: 0·54, 95% confidence interval [CI]: 0·30–0·98, *p* = 0·042) than women not experiencing violence. There was a trend towards increased IUD use among women experiencing physical IPV (adjusted RRR: 1·78, 95% CI: 0·91–3·41, *p* = 0·091) compared to those not experiencing physical IPV, but this did not reach statistical significance.

**Interpretation:**

Our findings suggest that women who experience physical IPV in India are less likely to use condoms and may be more likely to use IUDs than women without exposure to IPV. This research expands on prior findings suggesting higher uptake of women-controlled contraceptives among women contending with IPV in India.

**Research in context**

***Evidence before this study***

**Associations between intimate partner violence (IPV) and family planning use have been studied cross-sectionally in South Asia, and in India specifically, using both demographic nationwide data and data from parent trials. Prior findings have shown the association to vary based on type of IPV and type of contraceptive. In the literature, IPV has often been associated with decreased use of condoms and increased use of pills, suggesting that a woman's control over her contraceptive method may influence its use in the context of violence.**

***Added value of this study***

**We assessed the association between self-reported IPV and self-reported contraceptive use, by type, in a region of India with higher than average IUD use. In this study, we found a trend toward increased IUD use among women experiencing physical violence that did not reach statistical significance (*p* = 0.09). Comparing that trend to a decreased likelihood of condom use among women experiencing violence, found both in this study and in most prior, suggests that IPV may have a divergent association with IUDs as compared to condoms.**

***Implications of all the available evidence***

**Our study, along with prior evidence, suggests that the IUD, as a female-controlled contraceptive method, may be used more frequently by women experiencing IPV. Research into associations between IPV and family planning should continue to prioritize disaggregation by contraceptive type. Patient-centered clinical care, including family planning counseling and violence prevention programs, should similarly consider contraceptive methods by type.**

Alt-text: Unlabelled box

## Introduction

Intimate partner violence (IPV) affects one in three women worldwide [1]. In a recent nationwide survey, 23% of ever-married Indian women reported past-year physical IPV, while 5% of ever-married women nationwide reported past-year sexual IPV [2]. IPV affects women's reproductive health both directly, through injury and constrained access to healthcare and nutrition, and indirectly, through stress and trauma [1].

A woman's ability to control her own family planning choices is a critical aspect of her reproductive autonomy and key to increasing contraceptive use and decreasing unintended pregnancy. A 2015 assessment of abortion incidence in India found that nearly half of the country's 48 million pregnancies were unintended [3]. Unintended pregnancy increases the risk of maternal and infant morbidity and mortality, and many unintended pregnancies occur in the context of contraceptive non-use or failure [4]. A remaining challenge in meeting the reproductive health needs of women in India is ensuring a woman's ability to use contraception if desired [2,5].

In addition to contraceptive non-use or failure, IPV itself is an independent risk factor for unintended pregnancy globally [4]. Some studies have found that IPV is associated with increased use of contraception [6], [7], [8], [9], [10], [11], while others have shown that IPV is associated with decreased contraception use [12,13]. These divergent findings may be explained, in part, by differential effects of IPV on use of female-controlled contraceptive methods (e.g., intrauterine devices [IUDs]) and male-controlled methods (e.g., condoms) [14]. That is, we hypothesized that the use of contraceptive methods not dependent on a male partner's participation or approval may increase with IPV while methods that require male partner participation may decrease or remain the same. Furthermore, physical IPV and sexual IPV have both been associated with discordant contraceptive use (reported by a woman but not by her husband) among married Indian women, but these findings were not disaggregated by method [14]. When the association between IPV and modern contraceptive use is reported combining male and female controlled methods, the differential effects by method may be masked.

The purpose of this study is to assess the association between self-reported IPV and self-reported contraceptive use, by type, among married women in a cross-sectional study in rural Maharashtra, India.

## Methods

This is a cross-sectional study, conducted among women in rural Maharashtra between September 2018 and May 2019. Data were collected as part of the baseline questionnaire of the CHARM2 evaluation study (Counseling Husbands and wives to Achieve Reproductive health and Marital equity 2). CHARM2 is a gender synchronized family planning intervention for young couples; the study protocol has been previously published and described elsewhere in detail [15].

Briefly, eligible participants were recruited from randomly selected households in Pune District, rural Maharashtra. Eligible married couples were those where the wife was aged 18–29 years, able to speak Marathi, who had resided together for at least three months with plans to remain in their village for at least two years and were willing to participate in the intervention trial. Couples were ineligible if either spouse was cognitively impaired or if either spouse was sterilized or otherwise infertile.

Trained gender-matched research staff assessed eligibility and obtained written informed consent from couples prior to baseline survey participation, where couples were assessed separately in a private location. Baseline data on demographics, IPV exposure, and contraception use were collected from women participating in CHARM2 before the intervention was delivered. Because the primary outcome of this analysis was past 3-month contraceptive use, current analyses are limited to women who self-reported as not pregnant.

Baseline survey questions were based on measures from India's National Family Health Survey (NFHS-4) and other validated measures used in the original CHARM study [16]. The primary predictor, self-reported exposure to IPV (physical or sexual) within the past 12 months, was assessed by asking the woman if in the past 12 months she had experienced different forms of marital violence “often,” “sometimes,” or “not at all.” Physical IPV items ask whether a participant's husband had “slapped you,” “twisted your arm or pulled your hair,” “pushed you, shook you, or thrown something at you,” “kicked you, dragged you, or beat you up,” “choked you or tried to burn you on purpose,” or “threatened to attack you with a knife, gun, or any other weapon” in the past 12 months. Sexual IPV items ask whether a participant's husband had “physically forced you to have sexual intercourse with him even when you did not want to” or “forced you to perform sexual acts when you did not want to” in the past 12 months. These measures were aggregated to determine past 12-month exposure to a given form of IPV: a woman who answered “often” or “sometimes” to any of the physical or sexual IPV items, respectively, was coded as “yes” in a corresponding dichotomous variable about past 12 month physical or sexual IPV exposure. Women could report either or both forms of violence, and any woman reporting both forms was included as affirmative for both the physical IPV and the sexual IPV variables.

The outcome of interest, modern contraceptive use in the past 3 months, was based on a standard definition [17]. Modern contraceptive use was assessed by asking the woman, “Did you do something or use any method to delay or avoid getting pregnant in the past 3 months?” Women who answered yes were then asked which method(s) were used. For this analysis we evaluated use of oral contraceptive pills, condoms and IUDs, which are the only modern methods currently available on the public health system formulary in India and the most common methods in the study sample.

Demographic characteristics assessed included woman's age and age of partner, highest level of education completed by both spouses, caste (scheduled caste, scheduled tribe, other backward class, general caste), monthly family income, working outside the home to generate income in the past year (yes/no), age at marriage, parity, family composition (son(s), daughter(s), both, or no children), and family planning intentions (wants another child, does not want another child, cannot get pregnant, undecided).

The STROBE guidelines were used for reporting.

### Statistical analysis

The study sample size was determined to allow for assessment of the effectiveness of the CHARM2 intervention on modern contraceptive use, unintended pregnancy, and marital sexual violence, accounting for clustering.

Analyses were performed using Stata version 15·1 (StataCorp, College Station, TX, USA). Bivariate comparisons were analysed using a chi-squared test for categorical variables and ANOVA for continuous variables as appropriate. We performed crude and adjusted multinomial logistic regression models to assess the association between any IPV and contraceptive use by type. We also performed crude and adjusted multinomial logistic regression models to assess the association between IPV by type (physical or sexual) and contraceptive use by type. For each adjusted regression we used backward elimination on a set of baseline demographic variables to assess for inclusion in the model at a level of *p*<0·10. Crude and adjusted relative risk ratios (RRRs) and their 95% confidence intervals were calculated.

### Ethics

The study protocol was approved by the Institutional Review Boards at the National Institute for Research in Reproductive Health (NIRRH, Indian Council of Medical Research; Mumbai, India), Population Council (New York, NY, USA), and the University of California, San Diego (San Diego, CA, USA). All participants provided written informed consent prior to participation.

Our protocol included several procedures based on WHO guidelines for domestic violence research to prioritize the safety and minimize the vulnerability of participants reporting violence [18]. All women who participated in the baseline survey, regardless of reports of IPV, were notified verbally of local services for victims of domestic violence. Our trained staff also offered support to women reporting violence in forming safety plans should they choose to escape their situation. Any woman reporting life-threatening IPV was to be withdrawn from the study and referred directly to local services. Additional procedures to maximize the safety of participants reporting violence are detailed in our study protocol [15].

### Role of the funding source

No study sponsor had any role in study design; collection, analysis, or interpretation of data; or in writing this paper or the decision to submit for publication. The corresponding author had full access to all study data and had final responsibility for the decision to submit for publication. Funding was provided by the National Institutes of Health and the Bill & Melinda Gates Foundation (study #s NIH R01HD084453, 5K12HD001259; BMGF INV-002967).

## Results

Of the 1201 women enrolled in CHARM2, 1001 (83·3%) were not pregnant and provided complete answers to all study measures about IPV and contraceptive use and were therefore included in the present analysis.

On average, women were aged 24·1 years (Table 1). 379 women (37·9%) had used a modern contraceptive method in the past three months. Two-thirds (253) of those using contraception used condoms, 32 (8·4%) used pills and 89 (23·5%) used IUDs. One hundred and nine women (10·9%) reported experiencing physical IPV from their husbands in the past 12 months (Table 1) and 27 (2·7%) reported experiencing sexual IPV. Of the 114 women reporting violence in their marriage, thirty-six (31·6%) reported use of any modern contraceptive method.

**Table 1:** Demographic characteristics of non-pregnant married women (*n* = 1002)* living in rural Maharashtra: marriage characteristics, fertility preferences, and exposure to intimate partner violence.

	Total (*n* = 1001)	Past 3-month contraceptive use				p-value⁎⁎
		Any modern method (*n* = 379)*	Condoms (*n* = 253)	Pills (*n* = 32)	IUD (*n* = 89)	
	n (%)					
Age *mean (SD)*	24·1 (3·0)	24·6 (2·9)	24·8 (2·9)	24·4 (2·6)	24·0 (2·7)	<0·0001
Partner's age *mean (SD)*	29·6 (3·7)	30·1 (3·7)	30·3 (4·0)	29·4 (4·1)	29·7 (2·7)	<0·0001
*Highest education completed*						0·27
None or primary school	145 (14·5)	46 (12·1)	32 (12·7)	5(15·6)	9 (10·1)	
Secondary school or higher	856 (85·5)	333 (87·9)	221 (87·4)	27 (84·4)	80 (89·9)	
*Partner's education completed*						0·53
None or primary school	138 (13·8)	45 (11·9)	28 (11·1)	6 (18·8)	11 (12·4)	
Secondary school or higher	863 (86·2)	334 (88·1)	225 (88·9)	26 (81·2)	78 (87·6)	
*Caste*						0·0020
Scheduled caste/tribe or other backward class	321 (32·1)	110 (29·0)	86 (34·0)	5 (15·6)	18 (20·2)	
General	680 (67·9)	269 (71·0)	167 (66·0)	27 (84·4)	71 (79·8)	
Family monthly income, USD *mean (SD)*	265·9 (263·7)	281·6 (323·3)	296·4 (368·6)	229·2 (183·9)	263·6 (212·8)	0·11
*Working status in past year*						0·058
Works outside the home for income	556 (55·5)	212 (55·9)	130 (51·4)	21 (65·6)	59 (66·3)	
Does not work outside the home for income	445 (44·5)	167 (44·1)	123 (48·6)	11 (34·4)	30 (33·7)	
**Marriage characteristics and fertility preferences**						
Age at marriage, years *mean (SD)*	19·4 (2·4)	19·5 (2·3)	19·5 (2·4)	19·4 (2·5)	19·5 (2·2)	0·30
Parity *mean (SD)*	1·3 (0·7)	1·4 (0·7)	1·4 (0·7)	1·6 (0·7)	1·4 (0·6)	<0·0001
*Family composition*						<0·0001
Has no children	109 (10·9)	15 (4·0)	14 (5·5)	0	1 (1·1)	
Has only son(s)	385 (38·5)	158 (41·7)	103 (40·7)	10 (31·3)	41 (46·1)	
Has only daughter(s)	378 (37·8)	150 (39·6)	99 (39·1)	16 (50·0)	35 (39·3)	
Has both son(s) and daughter(s)	129 (12·9)	56 (14·8)	37 (14·6)	6 (18·8)	12 (13·5)	
*Family planning goals*						<0·0001
Wants another child	597 (59·6)	206 (54·4)	140 (55·3)	15 (46·9)	49 (55·1)	
Does not want another child	328 (32·8)	139 (36·7)	88 (34·8)	15 (46·9)	34 (38·2)	
Cannot get pregnant	0	0	0	0	0	
Undecided	76 (7·6)	34 (9·0)	25 (9·9)	2 (6·2)	6 (6·7)	
*Past 12-month gender-based violence*⁎⁎⁎						
Any IPV	114 (11·4)	36 (9·5)	18 (7·1)	3 (9·4)	15 (16·9)	0·087
Physical IPV	109 (10·9)	35 (9·2)	17 (6·7)	3 (9·4)	15 (16·9)	0·075
Sexual IPV	27 (2·7)	9 (2·4)	4 (1·6)	2 (6·3)	3 (3·4)	0·56

⁎ Four participants reported use of injectables only, and one participant reported use of emergency contraception only. Those five participants are included as users of “any modern method” but given their low frequencies were not included in the categorical outcome variable by method.

⁎⁎ p-value from chi^2^ test for categorical variables or ANOVA for continuous variables, as appropriate, for comparisons by type of modern contraceptive.

⁎⁎⁎ Women could report either or both forms of violence. 22 women reported both physical and sexual IPV, and they are included in each category.

There was no statistically significant association between any IPV and use of any modern method or between either type of IPV and use of any modern method. In contrast, when evaluating the association between type of IPV and contraceptive use by type, there was a divergent effect of physical violence on use of condoms as compared to IUDs (Table 2). After adjusting for covariates, women reporting physical IPV were significantly less likely to use condoms (adjusted RRR: 0·54, 95% CI: 0·30–0·98, *p* = 0·042) than women without exposure to violence. When adjusted for covariates, women who reported experiencing physical IPV were more likely to use IUDs (adjusted RRR: 1·78, 95% CI: 0·91–3·48, *p* = 0·091) than those who reported not experiencing physical IPV, but this trend did not reach statistical significance. Further adjusting each model for the other form of violence (i.e., adjusting for sexual violence in the physical violence models and adjusting for physical violence in the sexual violence models) did not meaningfully change results, and we had limited power to look at each type of violence independently (data not shown). As a sensitivity analysis, we performed the logistic regressions forcing all potential covariates into the models; these analyses did not meaningfully change results (data not shown).

**Table 2:** Unadjusted and adjusted multinomial logistic regression: associations between past 12-month physical IPV and sexual IPV with past 3-month contraceptive use in non-pregnant married women (*n* = 1002).

Intimate partner violence	Contraceptive use							
	Any modern method (*n* = 379)		Condoms (*n* = 253)		Pills (*n* = 32)		IUDs (*n* = 89)	
	Crude RRR (95% CI)	Adjusted RRR (95% CI)*	Crude RRR (95% CI)	Adjusted RRR (95% CI)#	Crude RRR (95% CI)	Adjusted RRR (95% CI)#	Crude RRR (95% CI)	Adjusted RRR (95% CI)#
Any IPV	0·73 (0·48–1·11)	0·77 (0·50–1·17)	0·53 (0·30–0·94)⁎⁎	0·54 (0·30–0·95)⁎⁎	0·72 (0·21–2·45)	0·82 (0·24–2·86)	1·41 (0·75–2·65)	1·61 (0·84–3·09)
Physical IPV	0·75 (0·49–1·15)	0·79 (0·51–1·22)	0·54 (0·30–0·96)⁎⁎	0·54 (0·30–0·98)⁎⁎	0·78 (0·23–2·64)	0·90 (0·26–3·15)	1·51 (0·80–2·85)	1·78 (0·91–3·41)
Sexual IPV	0·82 (0·36–1·84)	0·78 (0·35–1·78)	0·55 (0·17–1·76)	0·51 (0·16–1·63)	2·30 (0·49–10·81)	2·08 (0·43–10·15)	1·20 (0·33–4·39)	1·05 (0·28–3·94)

⁎ adjusted for woman's age, family composition, woman's education.

# adjusted for woman's age, family composition, and woman's caste.

⁎⁎ *p* < 0.05.

Oral contraceptive pills were used infrequently by women in this study. There was a trend to suggest that pills may be used more frequently by women reporting experiencing sexual IPV (adjusted RRR: 2·08, 95% CI: 0·43–10·15, *p* = 0·37).

## Discussion

Our analysis of the association between different forms of IPV and contraceptive use by type suggests that women who experience physical IPV in India are less likely to use condoms and more likely to use IUDs than women who do not experience physical IPV. Dasgupta and colleagues’ similar analysis found a positive association between physical IPV and condom use [7], but the negative association in our analysis aligns with most prior findings, in India and in a meta-analysis of the literature examining the effect of IPV on contraceptive use [12,19]. Given these divergent findings in the literature, Dasgupta's hypothesis that physical violence, occurring within the relationship but perhaps outside a couple's sexual encounters, may affect contraceptive use differently than sexual violence merits further research.

Our study's association between physical violence and decreased condom use should also be considered from a method-specific lens. Raj and colleagues’ analysis of national demographic surveys from India, Bangladesh, and Nepal found decreased condom use in pooled and country-specific models assessing the association between marital sexual violence and contraceptive use [8]. While our study found no significant association between sexual violence and condom use, our findings suggest that the male partner participation necessary for condom use may decrease its use across women experiencing all forms of violence.

An IUD, in contrast, may allow women experiencing IPV to control their own reproductive health securely and covertly, without requiring a male partner's knowledge or participation. As such, associations previously seen between IPV and discordant contraceptive use among married Indian couples might be further understood by assessing such use by family planning method [14]. The IUD's capacity for covert use may be especially salient in India, where decision-making around contraceptive use and birth-spacing are often influenced by a woman's husband or her in-laws [20,21]. As such, female-controlled contraceptive methods may grant women control over their family planning not only in situations of marital violence, but also within a patrilocal system, in which married couples live with or near the husbands’ parents.

IUD use is informed not only by perceptions about the degree of female control over the method, but also by method-specific perceptions of safety, efficacy, and comfort. Future research on IUD use in this population should consider previously reported hesitations held by providers and patients in India about the IUD, especially misconceptions regarding adverse effects (infertility, cancer, permanent adhesions) and fears about the insertion procedure [22,23].

Interpretation of pill use is limited due to the small numbers of pill users in this cohort, but there were non-significant point estimates suggesting that women reporting physical IPV may be less likely to use pills, while women reporting sexual IPV may be more likely to use pills. A positive association between sexual violence and pill use has been seen in an analysis of nationwide demographic data in India [8]. Past research has classified the pill as a female-controlled contraceptive method [14], but because pills may be discovered in the home, women's capacity to use them covertly may be limited. Further research is needed inclusive of a greater number of pill users to better understand how pill users perceive their control and autonomy over their chosen method, and whether that perceived control differs across forms of marital violence.

The primary strength of this study is that it examines associations between types of IPV and use of different types of contraceptive methods. By disaggregating modern contraception by type, our study highlights potentially meaningful differences in type of contraception used by women experiencing marital violence. In our study, disaggregating modern contraceptive use by type uncovered bidirectional relationships between violence and contraceptive types masked by a null overall finding when considering the association between IPV and all modern contraceptives. Prior null findings between forms of violence and non-disaggregated modern family planning use should be considered from this perspective [6,24]. In future research, assessing the relationship between violence and contraceptive by type will allow for more nuanced interpretations to guide clinical and policy interventions about violence prevention and family planning use.

This study has several limitations. As a cross-sectional analysis, the study cannot determine the temporality of associations. Further research is needed to examine the relationship between IPV and contraceptive use longitudinally. Also, in using baseline survey data, the analysis relies on women's self-reported responses to items about IPV and contraceptive use. In addition to recall bias, these reports are subject to social desirability bias and may have been underreported due to social stigma or, in the case of contraceptive use, overreported given the respondent's awareness of the survey being done in the context of a family planning intervention. The use of pills was relatively low in this study, as was the overall frequency of sexual IPV, and null findings should be considered within the context of limited power. Investigating similar associations with a larger sample size and/or cohort with higher frequency of each predictor and outcome will allow for more precision in assessing the trends we have found in this study. In addition, sterilized women were not included in this study, so we were unable to assess for associations between IPV and sterilization. Finally, while the IPV measures we used from NFHS do allow for comparisons to national data, the violence measures in NFHS are not as comprehensive as other instruments validated for use in India. For example, the Indian Family Violence and Control Scale would allow for a more nuanced assessment of IPV, including psychological and behavioural coercion, in future research [25].

These findings are most generalizable to young, married women in rural Maharashtra, India. In our population, physical and sexual violence were each less prevalent than in Indian nationwide data, while IUD use was more prevalent than in nationwide data [2]. Continued research in India and elsewhere is needed to better understand the relationship between intimate partner violence and contraceptive use in diverse populations.

Our findings highlight the importance of assessing the relationship between violence and contraceptive use by method. In this analysis, we unmasked divergent associations between violence and family planning when we disaggregated modern contraception by method. For research to most effectively guide interventions around IPV prevention and family planning, studies must continue to evaluate modern contraception by type and assess how IPV may be related to women's use of each method. Quality counselling programs and interventions to promote gender equity should similarly approach measuring family planning use by method. A method-specific approach to family planning research and programs is critical in ensuring women's reproductive autonomy.

## Declaration of Competing Interest

Ms. Chen has nothing to disclose. Dr. Silverman reports grants from National Institutes of Health and grants from Bill and Melinda Gates Foundation during the conduct of the study. Ms. Dixit has nothing to disclose. Dr. Begum has nothing to disclose. Dr. Ghule has nothing to disclose. Dr. Battala has nothing to disclose. Ms. Johns has nothing to disclose. Dr. Raj reports grants from National Institutes of Health and grants from Bill and Melinda Gates Foundation during the conduct of the study. Dr. Averbach reports grants from National Institutes of Health and grants from Bill and Melinda Gates Foundation during the conduct of the study.
